# Inactivation of Nitrite-Dependent Nitric Oxide Biosynthesis Is Responsible for Overlapped Antibiotic Resistance between Naturally and Artificially Evolved Pseudomonas aeruginosa

**DOI:** 10.1128/mSystems.00732-21

**Published:** 2021-09-21

**Authors:** Su-fang Kuang, Ding-yun Feng, Zhuang-gui Chen, Zhuo-zheng Liang, Juan-juan Xiang, Hui Li, Xuan-xian Peng, Tiantuo Zhang

**Affiliations:** a The Third Affiliated Hospital and State Key Laboratory of Bio-Control, School of Life Sciences, Southern Marine Science and Engineering Guangdong Laboratory (Zhuhai), Sun Yat-sen University, Guangzhou, People’s Republic of China; b Laboratory for Marine Fisheries Science and Food Production Processes, Qingdao National Laboratory for Marine Science and Technology, Qingdao, China; Arizona State University

**Keywords:** antibiotic resistance, nitric oxide, metabolomics, cefoperazone-sulbactam resistance, fumarate, NADH, nitrite, nitrate, sodium nitroprusside

## Abstract

Metabolic flexibility of Pseudomonas aeruginosa could lead to new strategies to combat bacterial infection. The present study used gas chromatography-mass spectrometry (GC-MS)-based metabolomics to investigate global metabolism in naturally and artificially evolved strains with cefoperazone-sulbactam (SCF) resistance (AP-R_CLIN-EVO_ and AP-R_LAB-EVO_, respectively) from the same parent strain (AP-R_CLIN_). Inactivation of the pyruvate cycle and nitric oxide (NO) biosynthesis was identified as characteristic features of SCF resistance in both evolved strains. Nitrite-dependent NO biosynthesis instead of an arginine-dependent NO pathway is responsible for the reduced NO, which is attributed to lower nitrite and electrons from the oxidation of NADH to NAD^+^ provided by the pyruvate cycle. Exogenous fumarate, NADH, nitrate, and nitrite promoted the NO level and thereby potentiated SCF-mediated killing. Unexpectedly, fumarate caused the elevation of nitrite, while nitrite/nitrate resulted in the increase of Cyt bc1 complex (providing electrons). These interesting findings indicate that the nitrite-dependent NO biosynthesis and the pyruvate cycle are mutual to promote NO metabolism. In addition, the NO-potentiated sensitivity to SCF was validated by NO donor sodium nitroprusside. These results reveal an endogenous NO-mediated SCF resistance and develop its reversion by metabolites in P. aeruginosa.

**IMPORTANCE** Infections with Pseudomonas aeruginosa have become a real concern among hospital-acquired infections, especially in cystic fibrosis patients and immunocompromised individuals. Control of the pathogen is challenging due to antibiotic resistance. Since bacterial metabolic state impacts sensitivity and resistance to antibiotics, exploring and disclosing bacterial metabolic mechanisms can be used to develop a metabolome-reprogramming approach to elevate bacterial sensitivity to antibiotics. Therefore, GC-MS-based metabolomics is used to explore the similarities and differences of metabolomes between naturally and artificially evolved cefoperazone-sulbactam (SCF)-resistant P. aeruginosa (AP-R_CLIN-EVO_ and AP-R_LAB-EVO_, respectively) from the same parent strain (AP-R_CLIN_). It identifies the depressed nitrite-dependent nitric oxide (NO) biosynthesis as the most overlapping characteristic feature between AP-R_CLIN-EVO_ and AP-R_LAB-EVO_. This is because the pyruvate cycle fluctuates, thereby generating fewer NADH and providing fewer electrons for nitrite-dependent NO biosynthesis than the control. Interestingly, exogenous fumarate, NADH, nitrate, and nitrite as well as NO donor sodium nitroprusside promote NO generation to elevate sensitivity to SCF. These results highlight the way to understand metabolic mechanisms of antibiotic resistance and explore metabolic modulation to combat the bacterial pathogen.

## INTRODUCTION

The emergence of bacterial antibiotic resistance continues to pose a significant public health problem worldwide in terms of mortality and economic loss. The rising antibiotic resistance is responsible for more than 2 million infections, with a death toll of 29,000 in the United States per annum and over 33,000 deaths and 874,000 disability-adjusted life years in Europe each year. These cause an attributable health care cost of more than $4.7 billion in the United States and $1.5 billion in direct and indirect costs in Europe (1–3). In this regard, antibiotic resistance represents one of the few challenges that attract global concerns for human and animal health and food safety (1). Thus, understanding antibiotic resistance mechanisms and developing efficient control approaches are an urgent need.

Recent reports have indicated that bacterial metabolic state impacts sensitivity and resistance to antibiotics, and thus, antibiotic-sensitive and antibiotic-resistant bacteria have differential metabolomes, termed antibiotic-sensitive and antibiotic-resistant metabolomes, respectively (4–6). The antibiotic-resistant metabolome can be reverted to antibiotic-sensitive metabolome by crucial biomarkers such as alanine, glucose, glutamate, which have been identified from comparison between antibiotic-resistant metabolome and antibiotic-sensitive metabolome (4, 7–12). Therefore, exploring and disclosing bacterial metabolic mechanisms can be used to develop a metabolome-reprogramming approach to elevate bacterial sensitivity to antibiotics.

Bacteria acquire antibiotic resistance via natural and artificial evolution. Artificial evolution is carried out in a condition-controlled laboratory, while natural evolution occurs in a condition-uncontrolled environment, such as in infected patients. Logically, more complex effects result from natural evolution than artificial evolution. The antibiotic effect is a useful clue to understand metabolic mechanisms of antibiotic resistance. Thus, exploring similarities and differences in metabolic changes between naturally and artificially evolved pathogens is especially important to identify antibiotic effect and remove nonantibiotic effect. Based on these clarified metabolic mechanisms mediated by the antibiotic effect, more efficient metabolome-reprogramming approaches can be developed to eliminate clinically antibiotic-resistant pathogens.

Pseudomonas aeruginosa is an opportunistic pathogen that is a leading cause of morbidity and mortality in cystic fibrosis (CF) patients and immunocompromised individuals (13). Treatment of P. aeruginosa infections can be difficult due to its natural and acquired resistance to antibiotics. Cefoperazone-sulbactam (SCF) is the first line of antibiotic used in clinical treatments. However, misuse and overuse of the drug have led to an increase in SCF resistance, and therefore, the risk of infections caused by drug-resistant bacteria has increased (14, 15). In recent years, metabolomic studies on P. aeruginosa have shown the diversity of experiments carried out, including comparison on metabolomes of isolates between patients suffering from cystic fibrosis and healthy people, characterization of compounds in the metabolome, identification of differential metabolic pathways and biomarkers on the function and dependence of this organism, and obtaining novel mechanistic information on polymyxin resistance (16–19). The understanding of the metabolic basis reveals that fumarate potentiates tobramycin susceptibility, while glyoxylate promotes tolerance (20). These studies are performed by artificially evolved strains or clinically evolved strains. However, information regarding the similarities and differences of metabolomes between an artificially evolved strain and a clinically evolved strain from the same parent strain is absent.

In this study, gas chromatography-mass spectrometry (GC-MS)-based metabolomics was used to explore the similarities and differences of metabolomes between naturally and artificially evolved SCF-resistant P. aeruginosa (AP-R_CLIN-EVO_ and AP-R_LAB-EVO_, respectively) from the same parent strain (AP-R_CLIN_). The depressed nitrite-dependent nitric oxide (NO) biosynthesis and decreased NO were identified as an importantly overlapping characteristic features of the two evolved SCF-resistant P. aeruginosa strains. NO promoted by fumarate, NADH, nitrate, and nitrite potentiated SCF-mediated killing, which was also detected in the presence of NO donor sodium nitroprusside. Interestingly, fumarate caused the elevation of nitrite, while nitrite/nitrate resulted in the increase of Cyt bc1 complex (providing electrons). This work demonstrates that inactivation of nitrite-dependent NO biosynthesis is an important mechanism for antibiotic resistance in AP-R_CLIN-EVO_ and AP-R_LAB-EVO_ and identifies a potential strategy to combat infection caused by SCF-resistant P. aeruginosa.

## RESULTS

### Phenotypes of AP-R_CLIN,_ AP-R_CLIN-EVO_, and AP-R_LAB-EVO_.

**FIG 1:**
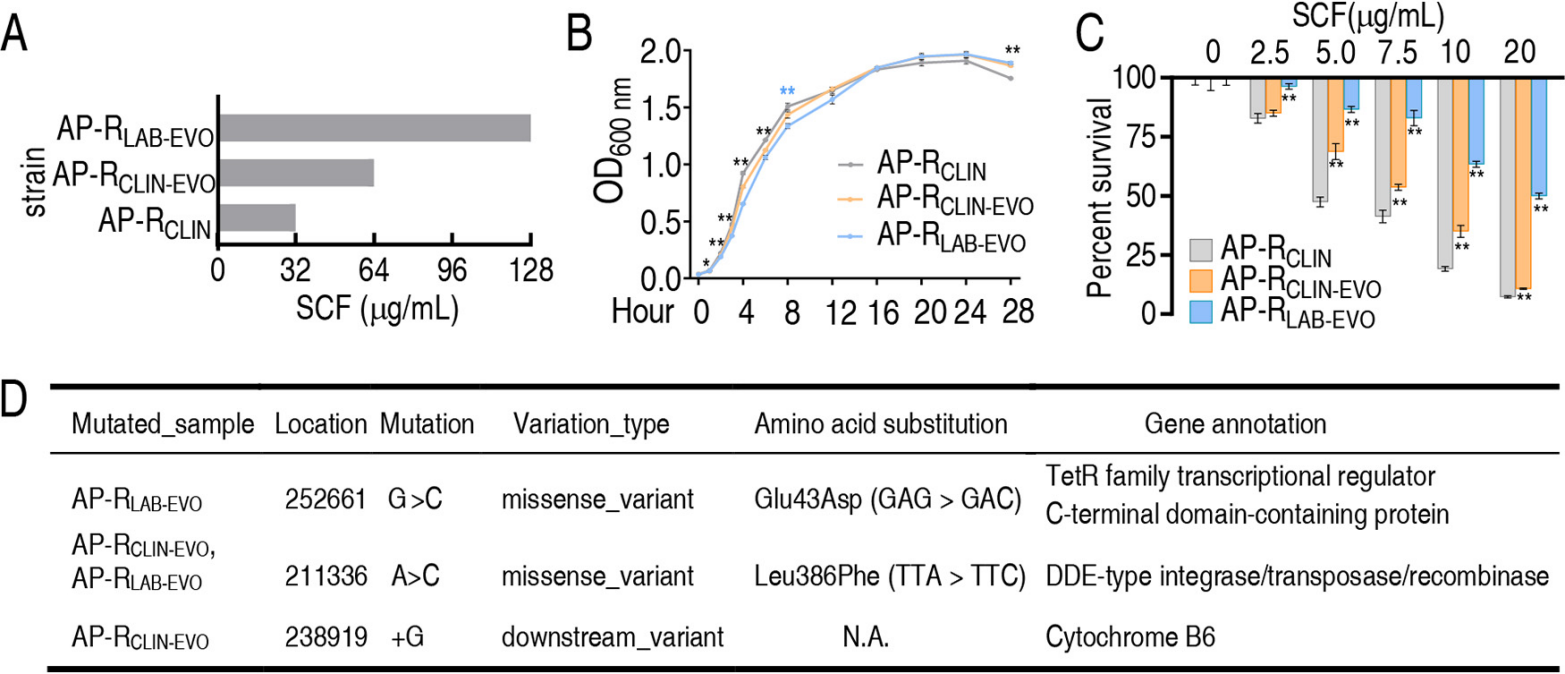
MIC, growth curve, survival, and gene mutation of AP-R_CLIN_, AP-R_CLIN-EVO_, and AP-R_LAB-EVO_. (A) MIC of the three bacterial strains to SCF. (B) Growth curve of the three bacterial strains in LB medium. (C) Survival of the three bacterial strains in the presence of the indicated concentration of SCF. (D) Mutations detected in whole-genome sequencing of AP-R_CLIN-EVO_ and AP-R_LAB-EVO_ compared to AP-R_CLIN_. Results in panels B and C are displayed as means ± SEM, and significant differences as determined by two-tailed Student’s *t* test are identified (*, *P* < 0.05; **, *P* < 0.01).

AP-R_CLIN_ and AP-R_CLIN-EVO_ were isolated from lower respiratory secretions of the same patient by using a bronchofiberscope at a 3-day interval. AP-R_LAB-EVO_ was obtained through sequential propagation of AP-R_CLIN_ in medium with SCF. The growth curve shows that slower growth at the earlier 12 h and more cultures at 48 h were detected for AP-R_CLIN-EVO_ and AP-R_LAB-EVO_ than AP-R_CLIN_ (Fig. 1A). The MICs of SCF for AP-R_CLIN_, AP-R_CLIN-EVO_, and AP-R_LAB-EVO_ were 32, 64, and 128 μg/ml, respectively (Fig. 1B). Consistently, survival capability was ranked as AP-R_LAB-EVO_ > AP-R_CLIN-EVO_ > AP-R_CLIN_ in an SCF dose-dependent manner (Fig. 1C). Whole-genome sequencing showed mutations of the gene encoding TetR family transcriptional regulator C-terminal domain-containing protein for AP-R_LAB-EVO_ and the gene encoding cytochrome B6 for AP-R_CLIN-EVO_, in addition to mutation of the gene encoding DDE-type integrase/transposase/recombinase in both strains (Fig. 1D). These gene mutations were confirmed in 20 clones of respective strains in comparison with 3 clones of AP-R_CLIN_ by PCR-based sequencing (see Table S1 in the supplemental material). Specifically, a missense downstream variant with amino acid substitutions G → C and A → C was detected in TetR family transcriptional regulator C-terminal domain-containing protein and DDE-type integrase/transposase/recombinase, respectively, while a downstream variant was found in cytochrome B6 (Fig. 1D and Fig. S1). TetR family transcriptional regulator participates in the transcriptional control of multidrug efflux pumps and other types of regulatory networks that underlie complex processes, such as homeostasis in metabolism (21, 22). Cytochrome B6 mediates electron transfer in the oxidative respiratory chain (23). The role of DDE-type integrase/transposase/recombinase is not clear. The information on the mutated genes motivated us to take metabolic changes as a key clue to explore the similarities and differences between AP-R_LAB-EVO_ and AP-R_CLIN-EVO_ for metabolic mechanisms of SCF resistance.

10.1128/mSystems.00732-21.1 FIG S1 Genome analysis of the mutation sequence between three bacterial strains. (A) Sequence alignment results of missense mutations in AP-R_LAB-EVO_ with TetR family transcriptional regulator C-terminal domain-containing protein (Glu43Asp, GAG → GAC). (B and C) Sequence alignment results of missense mutations in AP-R_LAB-EVO_ and AP-R_CLIN-EVO_ with DDE-type integrase/transposase/recombinase gene (Leu386Phe, TTA → TTC). (D) Sequence alignment results of missense mutations in AP-RCLIN-EVO with cytochrome B6 (+G). Download FIG S1, TIF file, 2.9 MB. Copyright © 2021 Kuang et al. 2021 Kuang et al. https://creativecommons.org/licenses/by/4.0/ This content is distributed under the terms of the Creative Commons Attribution 4.0 International license.

10.1128/mSystems.00732-21.4 TABLE S1 Strains isolated from a patient. Download Table S1, DOCX file, 0.01 MB. Copyright © 2021 Kuang et al. 2021 Kuang et al. https://creativecommons.org/licenses/by/4.0/ This content is distributed under the terms of the Creative Commons Attribution 4.0 International license.

### Differential metabolomes between naturally and artificially evolved strains.

**FIG 2:**
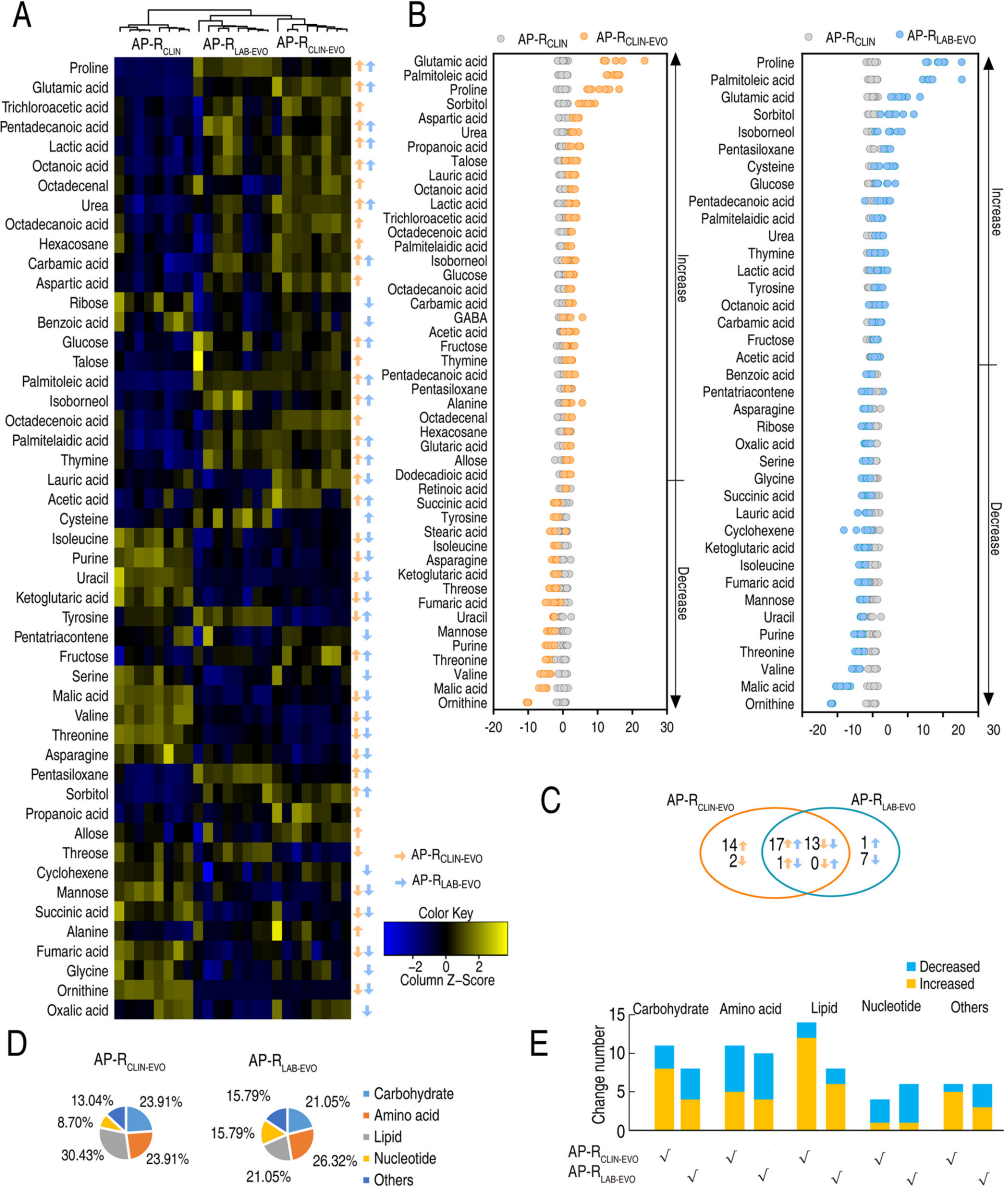
Differential metabolomes between naturally and artificially evolved strains. (A) Heat maps of differential abundance of metabolites (row). Yellow and blue indicate increase and decrease of the metabolites relative to the mean and SD of the row metabolite level, respectively (see color scale). (B) Z-score plots of differential abundances of metabolites based on control. Data from tested groups are separately scaled to the mean and SD of the control (AP-R_CLIN_). Each point represents one metabolite in one technical repeat and is colored by sample types. (C) Venn diagram showing the overlapping and unique differential metabolites between AP-R_CLIN-EVO_ and AP-R_LAB-EVO_. Decreased and increased metabolites are indicated with down and up arrows, respectively. (D) Categories of differential abundances of metabolites in AP-R_CLIN-EVO_ and AP-R_LAB-EVO_. (E) Increases and decreases in differential abundances of metabolites in panel D.

Antibiotic-resistant bacteria have antibiotic-resistant metabolomes (7). To explore the metabolic mechanisms and crucial metabolites of SCF resistance between AP-R_CLIN_ and AP-R_CLIN-EVO_ or AP-R_LAB-EVO_, a GC-MS-based metabolomics methodology was used to characterize the metabolic profiles of the three strains. Four biological samples with two technical replicates were examined in each group, yielding a total of 24 data sets. A total of 230 aligned individual peaks were obtained from each sample (Fig. S2A). The correlation coefficient between technical replicates varied between 0.990 and 0.999, demonstrating the reproducibility of the data (Fig. S2B). After removal of the internal standard ribitol and any known artificial peaks, 70 metabolites were determined (Fig. S2C). Among them, 22.86%, 24.29%, 30.00%, 12.86%, and 22.86% were carbohydrates, amino acids, lipids, nucleotides, and others, respectively (Fig. S2D). Compared with the AP-R_CLIN_ group using a Kruskal-Wallis test (*P* < 0.05), the AP-R_CLIN-EVO_ group and AP-R_LAB-EVO_ group exhibited 46 and 38 differentially abundant metabolites, respectively (Fig. 2A). The Z-scores varied between −10.09 and 14.96 in AP-R_CLIN-EVO_ group and −11.61 and 19.30 in AP-R_LAB-EVO_ group (Fig. 2B). Among these differential metabolites, 31 overlapped between the two groups and 16 and 8 existed only in the AP-R_CLIN-EVO_ group and AP-R_LAB-EVO_ group, respectively (Fig. 2C). Specifically, among the 31 overlapping metabolites, 17 (glutamic acid, palmitoleic acid, proline, sorbitol, urea, lauric acid, octanoic acid, lactic acid, palmitelaidic acid, isoborneol, glucose, carbamic acid, acetic acid, fructose, thymine, pentadecanoic acid, and pentasiloxane) and 13 (succinic acid, tyrosine, isoleucine, asparagine, ketoglutaric acid, fumaric acid, uracil, mannose, purine, threonine, valine, malic acid, and ornithine) were elevated and decreased, respectively, in two groups. These differential abundances of metabolites are distributed as 23.91% and 21.05% carbohydrate, 23.91% and 26.32% amino acid, 30.43% and 21.05% lipid, 8.70% and 15.79% nucleotide, and 13.04% and 15.79% other metabolites in AP-R_CLIN-EVO_ and AP-R_LAB-EVO_, respectively (Fig. 2D). The number and distribution of these differential metabolites at abundance are shown in Fig. 2E; almost carbohydrates and fatty acids were upregulated. These results indicate that the naturally and artificially evolved strains induced by SCF have similarities and differences in metabolism.

10.1128/mSystems.00732-21.2 FIG S2 Metabolomic profiling of AP-R_CLIN_, AP-R_CLIN-EVO_, and AP-R_LAB-EVO_. (A) Representative total ion current chromatogram. (B) Abundance of metabolites quantified in samples over two technical replicates is shown. Pearson correlation coefficient between technical replicates varies between 0.990 and 0.999. This plot shows the two replicates with the weakest correlation, 0.997. (C) Heat map of unsupervised hierarchical clustering of all metabolites (row). Yellow and blue indicate increase and decrease of the metabolites scaled to mean and standard deviation of row metabolite level, respectively (see color scale). (D) Category of all of the identified metabolites. Download FIG S2, TIF file, 2.3 MB. Copyright © 2021 Kuang et al. 2021 Kuang et al. https://creativecommons.org/licenses/by/4.0/ This content is distributed under the terms of the Creative Commons Attribution 4.0 International license.

### Differential and shared metabolic pathways and biomarkers between naturally and artificially evolved strains.

**FIG 3:**
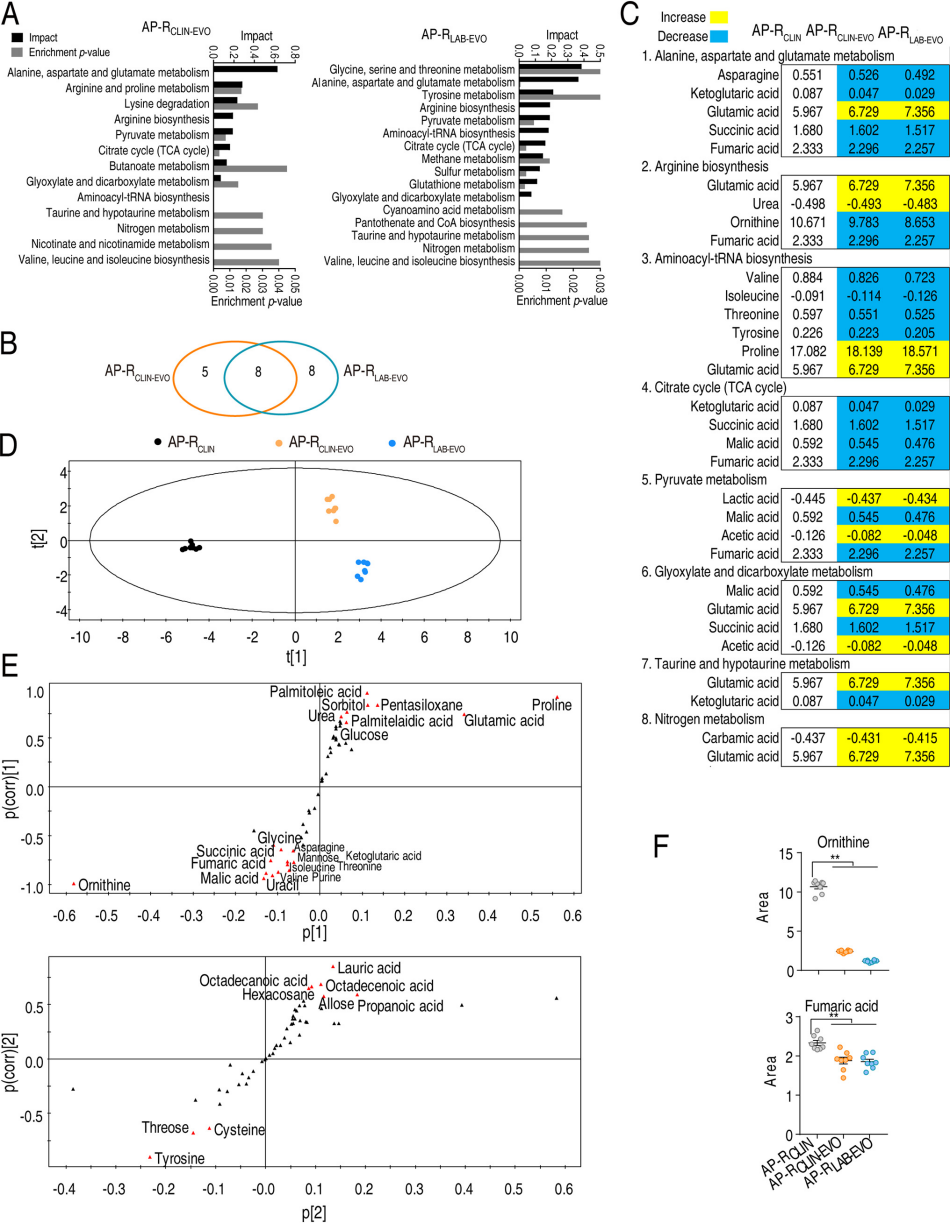
Overlapping pathways, metabolites, and multivariate data analysis between AP-R_CLIN-EVO_ and AP-R_LAB-EVO_. (A) Enriched pathways by differential abundances of metabolites. (B) Venn diagram showing overlapping and unique differential pathways. (C) Integrative analysis of metabolites in significantly enriched pathways. Yellow and blue indicate increase and decrease of metabolites, respectively. Number shows the relative value of differential abundance of metabolites. (D) PCA of AP-_CLIN_, AP-R_CLIN-EVO_, and AP-R_LAB-EVO_. Each dot represents the technical replicate of samples in the plot. (E) S-plot generated from OPLS-DA. Predictive component p[1] and correlation p(corr)[1] differentiate AP-R_CLIN_ and AP-R_LAB-EVO_ from AP-R_CLIN-EVO_. Predictive component p[2] and correlation p(corr)[2] separate AP-R_CLIN_ from AP-R_LAB-EVO_ and deviations of AP-R_CLIN-EVO_. The triangle represents metabolites in which candidate biomarkers are marked. (F) Scatter diagram of biomarkers ornithine identified by p[1] and p(corr)[2] and arginine identified by p[2] and p(corr)[2]. *, *P* < 0.05; **, *P* < 0.01.

Changes in metabolic pathways are directly related to bacterial resistance (9, 10). Therefore, enrichment of differentially metabolic pathways is a key to understanding the metabolic mechanisms induced by SCF. When these differential abundances of metabolites were analyzed, 13 and 16 metabolic pathways were found to be separately enriched in both the AP-R_CLIN-EVO_ group and AP-R_LAB-EVO_ group (Fig. 3A). Among them, 8 metabolic pathways (alanine, aspartate, and glutamate metabolism, arginine biosynthesis, pyruvate metabolism, tricarboxylic acid (TCA) cycle, glyoxylate and dicarboxylate metabolism, aminoacyl-tRNA biosynthesis, taurine and hypotaurine metabolism, and nitrogen metabolism) overlapped in the two groups. However, 5 and 8 metabolic pathways were enriched only in the AP-R_CLIN-EVO_ group and AP-R_LAB-EVO_ group, respectively (Fig. 3B). Interestingly, among the overlapping metabolic pathways, alanine, aspartate, and glutamate metabolism and arginine biosynthesis were the two most impacted pathways. In the two pathways, the decreased ornithine, ketoglutaric acid, and fumaric acid and the elevated urea and glutamic acid overlapped between the two strains (Fig. 3C). These results indicate that the similarities are greater than the differences between AP-R_CLIN-EVO_ and AP-R_LAB-EVO_.

Pattern recognition is an efficient tool to identify biomarkers in metabolomics analysis. Thus, orthogonal partial least square discriminant analysis (OPLS-DA) was carried out to recognize the sample patterns of metabolomes as shown in Fig. 3D. Component t[1] separated AP-R_CLIN_ and AP-R_LAB-EVO_ from AP-R_CLIN-EVO_, while component t[2] differentiated AP-R_LAB-EVO_ and AP-R_CLIN-EVO_ from AP-R_CLIN_. Furthermore, discriminating variables were shown by S-plot. In the plots of predictive correlation between p[1] and p(corr)[1] and p[2] and p(corr)[2], the red triangle indicates the differential metabolites that have larger weights (less than −0.05 or greater than 0.05) and higher relevance (less than −0.5 or greater than 0.5). The analysis led to the identification of 21 (asparagine, fumaric acid, glucose, glutamic acid, glycine, isoleucine, ketoglutaric acid, malic acid, mannose, ornithine, palmitelaidic acid, palmitoleic acid, pentasiloxane, proline, purine, sorbitol, succinic acid, threonine, uracil, urea, and valine) and 9 (allose, cysteine, hexacosane, lauric acid, octadecanoic acid, octadecenoic acid, propanoic acid, threose and tyrosine) biomarkers in p[1] and p(corr)[1] and p[2] and p(corr)[2], respectively (Fig. 3E). Among them, no metabolites overlapped. We paid attention to the biomarkers identified by p[1] and p(corr)[1] since the analysis differentiated the two tested groups from the control, where the abundances of fumaric acid, ornithine, glycine, isoleucine, ketoglutaric acid, malic acid, and uracil are downregulated, while the abundances of glucose, palmitoleic acid, pentasiloxane, and proline are upregulated (Fig. 3F and Fig. S3). Among them, fumaric acid and ornithine is related to NO formation. These results show that NO metabolism is related to the drug resistance evolution in both AP-R_LAB-EVO_ and AP-R_CLIN-EVO_.

10.1128/mSystems.00732-21.3 FIG S3 Scatter diagram of biomarkers identified by S-plot. Download FIG S3, TIF file, 1.8 MB. Copyright © 2021 Kuang et al. 2021 Kuang et al. https://creativecommons.org/licenses/by/4.0/ This content is distributed under the terms of the Creative Commons Attribution 4.0 International license.

### Nitrite-dependent NO biosynthesis instead of arginine-dependent NO pathway is responsible for NO decrease in AP-R_CLIN-EVO_ and AP-R_LAB-EVO_.

**FIG 4:**
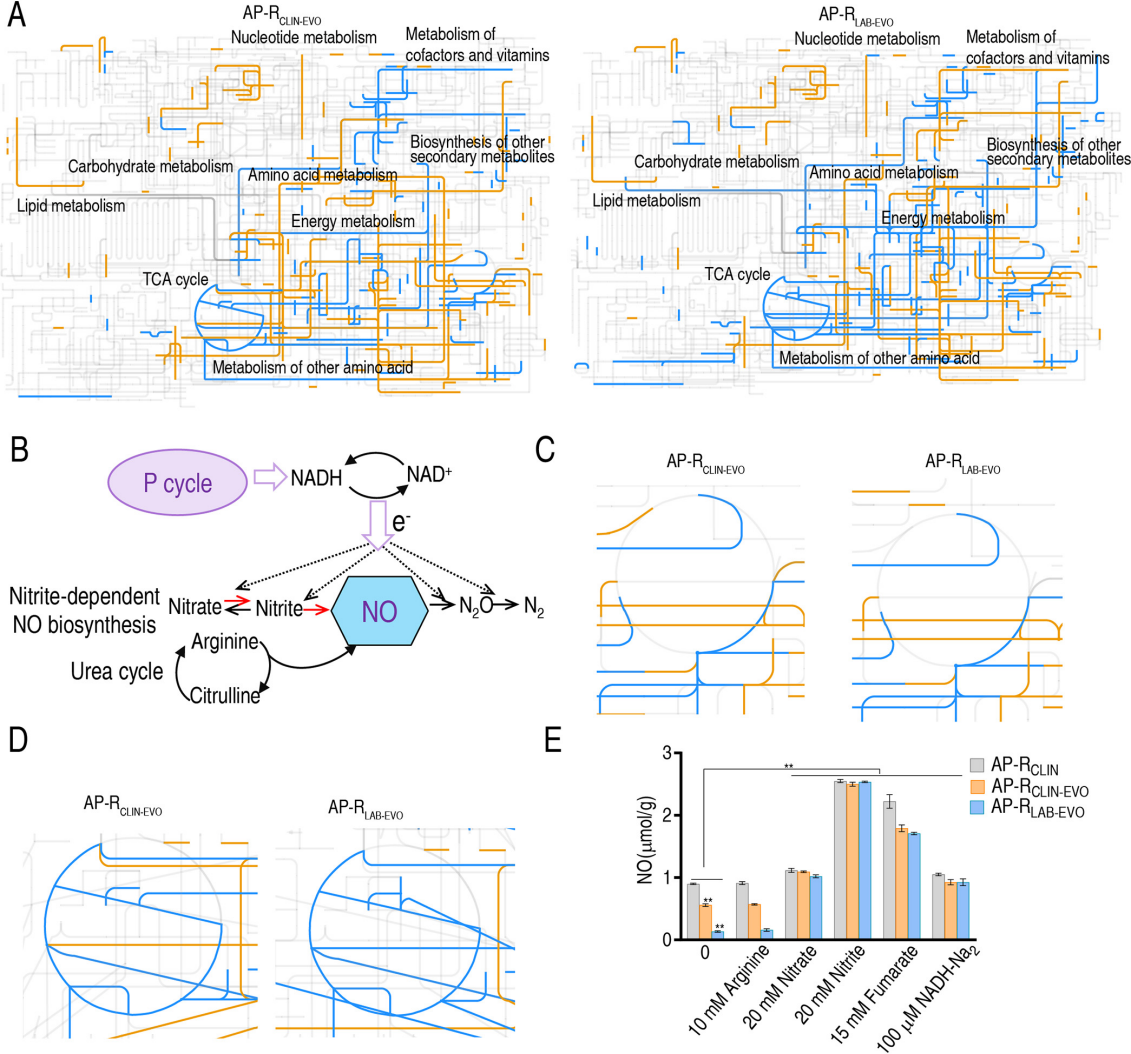
iPath analysis and measurement of NO. (A) iPath analysis. (B) Outline for NO biosynthesis. The dotted line represents the direction of electron transfer. (C) Enlarged map for urea cycle based on data in panel A. (D) Enlarged map for TCA cycle based on data in panel A. (E) NO level in the absence or presence of arginine, fumarate, nitrate, and nitrite. Results are displayed as means ± SEM, and significant differences as determined by two-tailed Student’s *t* test are identified (*, *P* < 0.05; **, *P* < 0.01).

Interactive Pathways (iPath) was used to provide global overview maps for a better insight into the similarities between AP-R_CLIN-EVO_ and AP-R_LAB-EVO_ compared to AP-R_CLIN_; in Fig. 4A, yellow and blue lines represent increased and decreased pathways, respectively, in AP-R_CLIN-EVO_ (left) and AP-R_LAB-EVO_ (right) Generally, the two global overview maps were similar, although some differences were characterized, including local higher carbohydrate metabolism, nucleotide metabolism, and other amino acid metabolism in AP-R_CLIN-EVO_ than in AP-R_LAB-EVO_. It is known that NO biosynthesis is attributable to two metabolic pathways, the l-arginine-dependent NO pathway and nitrite-dependent NO biosynthesis. In the l-arginine-dependent NO pathway, the oxidation of l-arginine is catalyzed to nitric oxide and l-citrulline by nitric oxide synthase (NOS). In nitrite-dependent NO biosynthesis of P. aeruginosa, conversion of nitrite to NO in dissimilatory denitrification is catalyzed by the enzyme nitrite reductase (NiR), where an electron is provided by the pyruvate cycle, a recently clarified cycle providing respiratory energy in bacteria, and by oxidation of NADH back to NAD^+^ (Fig. 4B). The urea cycle, containing the l-arginine-dependent NO pathway, and the TCA cycle were similar between the two strains (Fig. 4C and D). We further showed that NO level is ranked from low to high: AP-R_LAB-EVO_ < AP-R_CLIN-EVO_ < AP-R_CLIN_ (Fig. 4E), which was consistent with the MIC (Fig. 1A). To explore which pathway, the nitrite-dependent or l-arginine-dependent NO biosynthesis pathway, is responsible for the decreased NO, exogenous arginine, nitrate, nitrite, fumarate, and NADH were tested. Nitrate, nitrite, fumarate, and NADH promoted NO level but arginine did not. The promotion by nitrate, nitrite, fumarate, and NADH led to the same NO level as control AP-R_CLIN_ did (Fig. 4E). These results indicate that nitrite-dependent NO biosynthesis is responsible for NO decrease in AP-R_CLIN-EVO_ and AP-R_LAB-EVO_.

### Inactivation of nitrite-dependent NO biosynthesis contributes to NO decrease in AP-R_CLIN-EVO_ and AP-R_LAB-EVO_.

**FIG 5:**
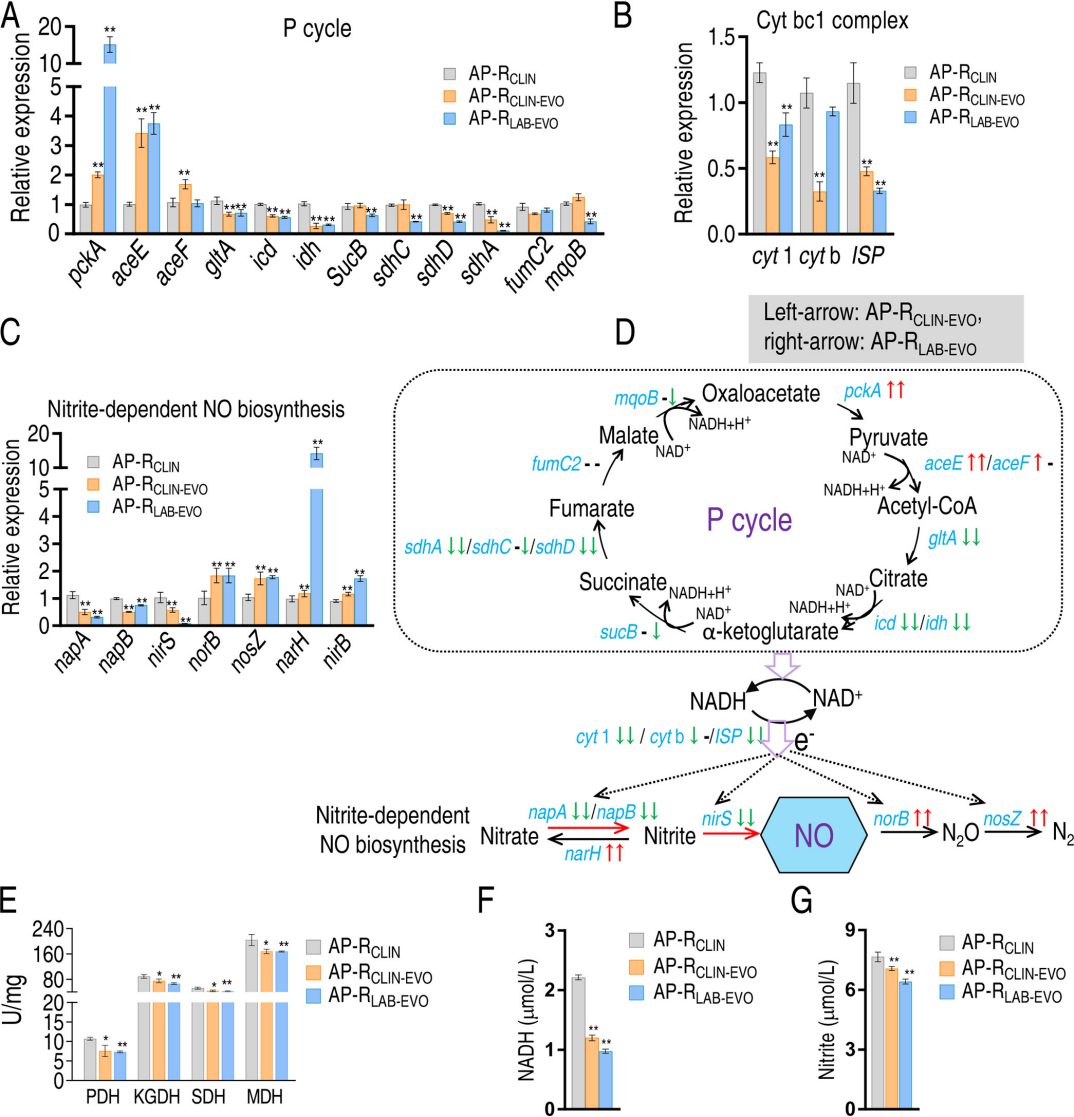
P cycle-mediated NO reduction in nitrite-dependent NO biosynthesis. (A to C) qRT-PCR for gene expression of the P cycle (A), cytb1 complex (B), and NO-dependent NO biosynthesis (C). (D) Outline for changes in gene expression of NO biosynthesis based on data in panel A. Blue, genes detected; gray, genes undetected. (E) Activity of enzyme activity in the P cycle. (F) NADH level. (G) Nitrite level. Results in panels A to C and E to G are displayed as means ± SEM, and significant differences as determined by two-tailed Student’s *t* test are identified (**, *P* < 0.01).

The above-described results motivated us to demonstrate that NO decrease is attributable to nitrite-dependent NO biosynthesis and fluctuation of the P cycle, since the P cycle provides electrons for nitrite-dependent NO biosynthesis via Cyt bc1 complex. To do this, quantitative real-time PCR (qRT-PCR) was used to detect expression of genes in the P cycle, Cyt bc1 complex, and nitrite-dependent NO biosynthesis. Among 12, 3, and 6 genes detected in the P cycle, Cyt bc1 complex, and nitrite-dependent NO biosynthesis, respectively, most were downregulated except for upregulated *pckA* and *aceE/F* of both strains and *mcpB* of AP-R_LAB-EVO_ in the P cycle (Fig. 5A), unchanged *cytb* of AP-R_LAB-EVO_ in Cyt bc1 complex (Fig. 5B), and upregulated *norB*, *nosZ*, *narH*, and *nirB* of nitrite-dependent NO biosynthesis of both strains in nitrite-dependent NO biosynthesis (Fig. 5C). In the P cycle and Cyt bc1 complex, almost all genes were reduced in expression, suggesting that limited electrons are provided. In nitrite-dependent NO biosynthesis, the upregulated genes work for NO degradation (*norB* and *nosZ*) and transformation of nitrite to nitrate and NH_4_^+^ (*narH* and *nirB*, respectively), while the downregulated genes play a role in NO generation (*nirS*, *napA*, and *napB*). These results suggest that NO decrease is attributable to decreased electron donor NADH and decreased NO generation and elevated NO degradation in AP-R_CLIN-EVO_ and AP-R_LAB-EVO_ compared to AP-R_CLIN_ (Fig. 5D). Activity of enzymes supported that the P cycle is inactivated (Fig. 5E). Consistently, lower NADH and nitrite were measured in AP-R_CLIN-EVO_ and AP-R_LAB-EVO_ than AP-R_CLIN_ (Fig. 5F and G). These results indicate that inactivation of nitrite-dependent NO biosynthesis is responsible for NO decrease in AP-R_CLIN-EVO_ and AP-R_LAB-EVO_.

### Fumarate and NADH promote nitrite-dependent NO biosynthesis and elevate bacterial sensitivity to SCF.

**FIG 6:**
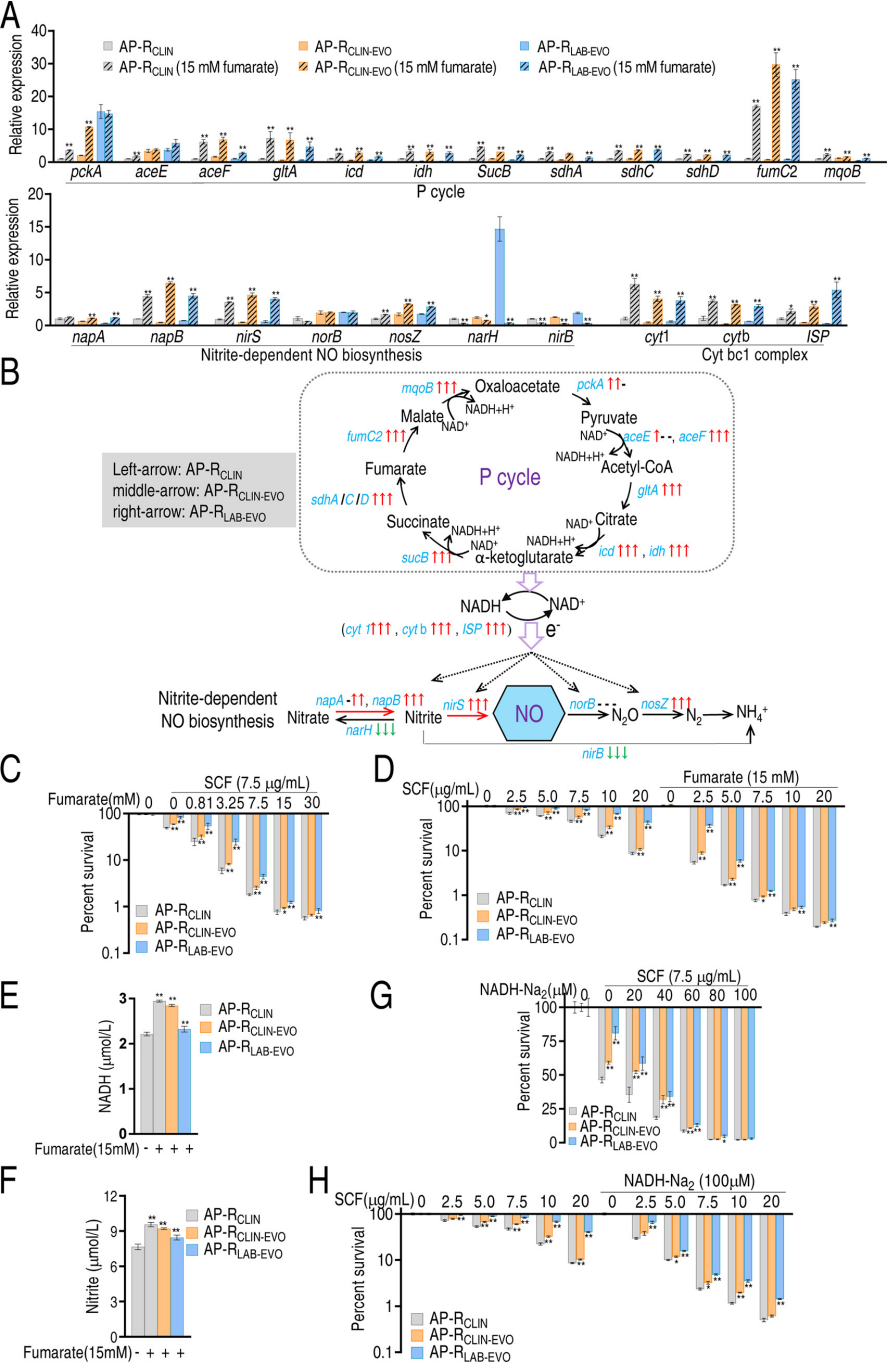
Effect of fumarate-based NO biosynthesis on SCF-mediated killing. (A) qRT-PCR for expression of genes encoding the P cycle and nitrite-dependent NO in the presence of fumarate. (B) Outline for changes based on data in panel A. Blue, genes detected; gray, genes undetected. (C and D) Percent survival of the three strains at the indicated concentrations of fumarate (C) and SCF (D). (E) NADH in the presence of fumarate. (F) Nitrite in the presence of fumarate. (G and H) Percent survival of the three strains in the indicated concentrations of NADH (G) and SCF (H). Results in panels A and C to G are displayed as means ± SEM, and significant differences as determined by two-tailed Student’s *t* test are identified (*, *P* < 0.05; **, *P* < 0.01).

To demonstrate that the decreased electron donor NADH is attributable to the fluctuated P cycle, fumarate was used to promote the P cycle, Cyt bc1 complex, and nitrite-dependent NO biosynthesis. qRT-PCR showed that all genes generally are upregulated except for downregulated *narH* and *nirB* and unchanged *norB* (Fig. 6A). Thus, the P cycle was activated to provide more electrons and thereby NO generation and nitrite transformation to nitrate and NH_4_^+^ were promoted and inhibited, respectively (Fig. 6B). Exogenous fumarate promoted SCF-mediated killing to AP-R_CLIN_, AP-R_CLIN-EVO_, and AP-R_LAB-EVO_ in a dose-dependent manner (Fig. 6C). The killing was elevated with the increasing dose of SCF (Fig. 6D). Consistently, exogenous fumarate led to elevation of NADH and nitrite levels (Fig. 6E and F). When exogenous fumarate was replaced with exogenous NADH, the SCF-mediated killing was also increased with the increasing dose of NADH and SCF (Fig. 6G and H). These results indicate that the fluctuated P cycle causes the decreased electron donor for NO generation due to the reduction of NADH.

### Nitrate and nitrite promote nitrite-dependent NO biosynthesis and elevate bacterial sensitivity to SCF.

**FIG 7:**
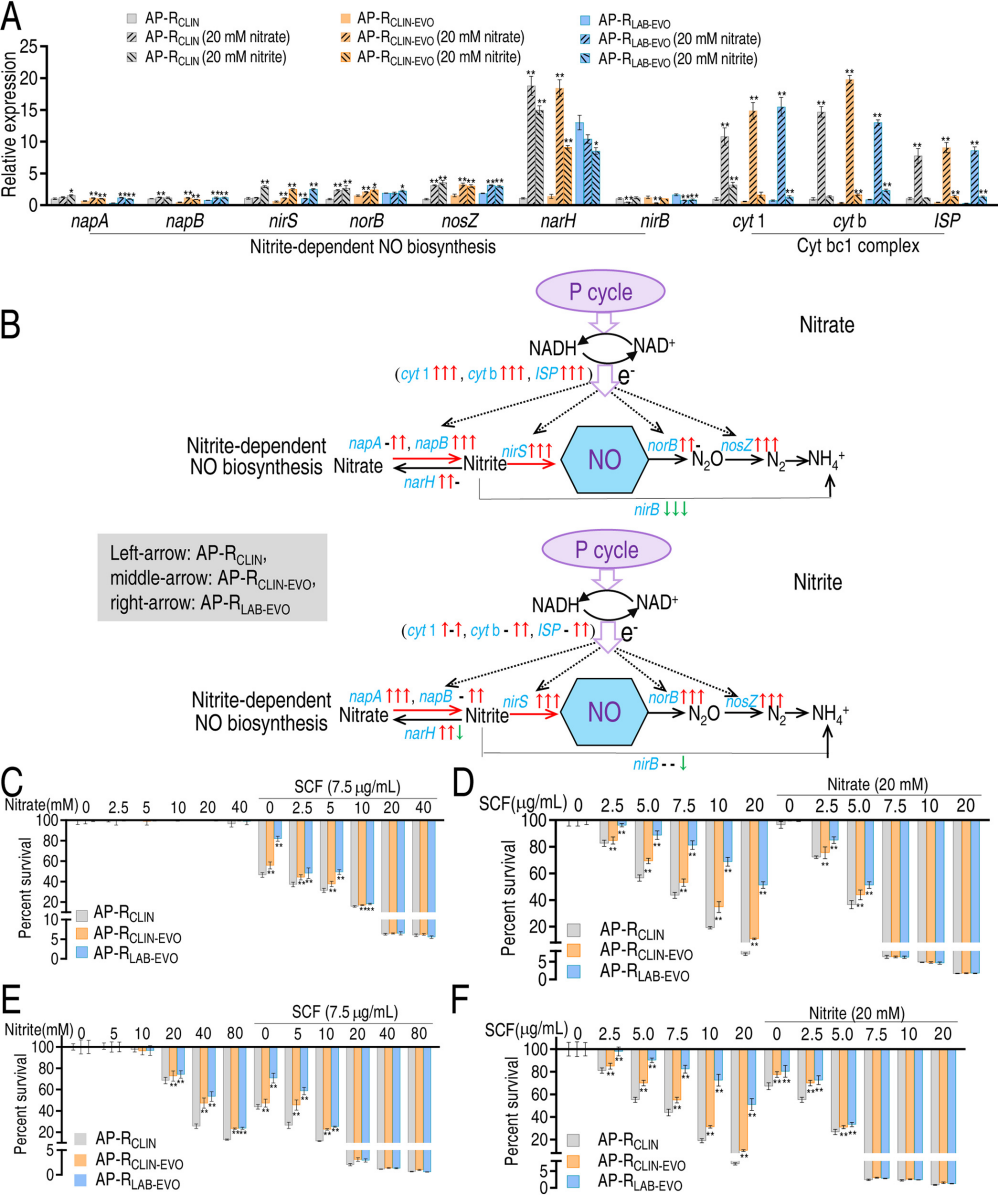
Effect of nitrite-based NO biosynthesis on SCF-mediated killing. (A) qRT-PCR for expression of genes encoding nitrate-based NO biosynthesis enzymes in the presence of nitrate or nitrite. (B) Outline for changes in gene expression of NO biosynthesis based on data in panel A in the presence of nitrate or nitrite. The down arrow represents downregulation, and the up arrow represents upregulation. The left arrow and right arrow represent AP-_CLIN-EVO_ and AP-_LAB-EVO_, respectively. The black arrow shows the same changes in gene expression in the presence of nitrate or nitrite; the purple arrow represents changes in gene expression only in the presence of NaNO_3_. Blue is used for gene names not found. (C and D) Percent survival of the three strains at the indicated concentrations of nitrate (C) and SCF (D). (E and F) Percent survival of the three strains at the indicated concentrations of nitrite (E) and SCF (F). Results in panels A and C to F are displayed as means ± SEM, and significant differences as determined by two-tailed Student’s *t* test are identified (*, *P* < 0.05; **, *P* < 0.01).

We supposed that nitrate and nitrite could promote nitrite-dependent NO biosynthesis since nitrate and nitrite are substrates of the nitrate-nitrite-nitric oxide pathway. To demonstrate this, the three strains were cultured in M9 medium with nitrate or nitrite. qRT-PCR was used to measure expression of genes in the nitrate-nitrite-nitric oxide pathway. Exogenous nitrate and nitrite promoted expression of all genes (partly unchanged) except for *nirB*, which was reduced (Fig. 7A and B). Nitrate elevated SCF-mediated killing efficacy in a dose-dependent manner, where no difference was detected between AP-R_CLIN_ and AP-R_CLIN-EVO_ or AP-R_LAB-EVO_ when 20 or 40 mM nitrate plus 7.5 μg/ml SCF was used (Fig. 7C). SCF-mediated killing was increased in a dose-dependent manner. Nitrate promoted the killing efficacy with increasing nitrate doses. There was a difference in the killing efficacy between AP-R_CLIN_ and AP-R_CLIN-EVO_ or AP-R_LAB-EVO,_ but no difference was found when 7.5 to 20 μg/ml SCF and 20 mM nitrate were used (Fig. 7D). Similar results were obtained when nitrate was replaced with nitrite (Fig. 7E and F). These data suggest that enhancement of the nitrate-nitrite-nitric oxide pathway reverts bacterial resistance to SCF.

### NO plays a role in reverting bacterial resistance to SCF.

**FIG 8:**
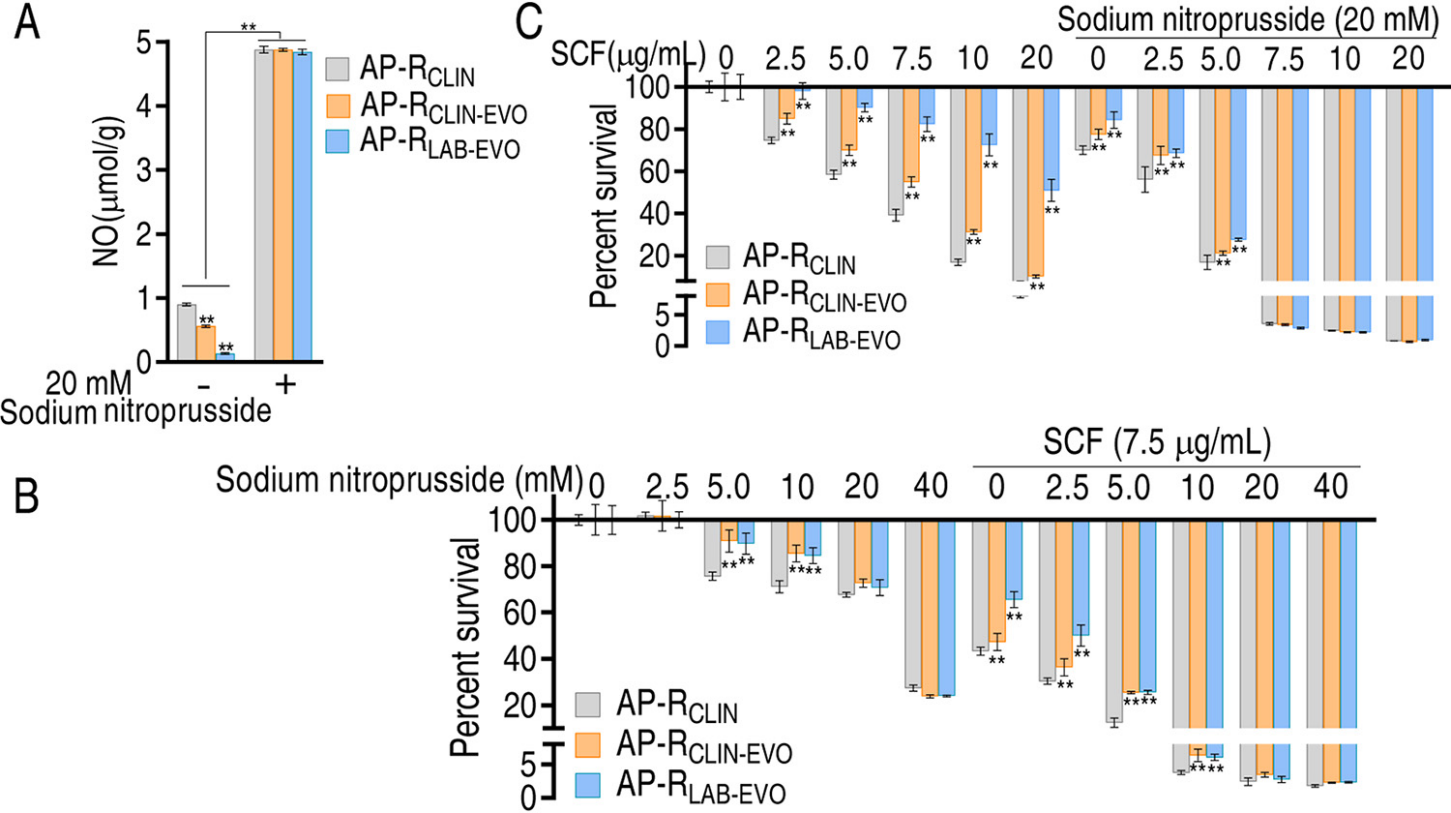
Effect of promoting inducible NO on SCF-mediated killing. (A) NO level in the presence of sodium nitroprusside. (B and C) Percent survival of the three strains at the indicated concentrations of sodium nitroprusside (B) or SCF (C). Results are displayed as means ± SEM, and significant differences as determined by two-tailed Student's *t* test are identified (**, *P* < 0.01).

Logically, since enhancement of nitrite-dependent NO biosynthesis promotes the NO level and reverts bacterial resistance to SCF, sodium nitroprusside, a nitric oxide donor, should have the same action. Therefore, nitrate or nitrite was replaced with sodium nitroprusside. Sodium nitroprusside promoted the NO level of AP-R_CLIN_, AP-R_CLIN-EVO_ and AP-R_LAB-EVO_, where no difference was detected among the three strains (Fig. 8A). Sodium nitroprusside showed a bactericidal effect in a dose-dependent manner, but synergistic use of sodium nitroprusside and SCF enhanced the effect with increasing sodium nitroprusside from 2.5 to 10 mM plus 7.5 μg/ml of SCF. The effect was the similar when 20 to 40 mM sodium nitroprusside was used (Fig. 8B). Meanwhile, we showed that the SCF-mediated killing efficacy was related to the dose of sodium nitroprusside used, and no difference was detected between SCF at 7.5 to 20 μg/ml and 20 mM nitroprusside (Fig. 8C). These results support the conclusion that NO reduction plays a role in the resistance to SCF.

## DISCUSSION

Metabolic flexibility of P. aeruginosa could lead to new strategies to combat bacterial infection (24). The present study explored metabolic mechanisms of SCF resistance in P. aeruginosa evolved *in vivo* and *in vitro*. Whole-genome sequencing showed that mutations of two genes were separately identified in AP-R_CLIN-EVO_ and AP-R_LAB-EVO_ compared to AP-R_CLIN_, where one overlapped. Among them, TetR family transcriptional regulator C-terminal domain-containing protein and cytochrome *b*_6_ play a role in metabolism (21–23). These results not only indicate that AP-R_CLIN_ is a parent strain of AP-R_CLIN-EVO_ and AP-R_LAB-EVO_ but also provide a clue to investigate SCF resistance mechanisms from the perspective of metabolism.

It has been shown that many of the key adaptations that arise in P. aeruginosa during infection are centered on the core metabolism (24). The metabolomes of wild-type P. aeruginosa strain K (polymyxin B MIC, 1 mg/liter) and its paired *pmrB* mutant strains, PAKpmrB6 and PAKpmrB12 (polymyxin B MICs of 16 mg/liter and 64 mg/liter, respectively), show that the decreased phospholipid level is associated with polymyxin resistance (19). The complex and dynamic interactions of multiple cellular pathways are associated with the polymyxin mode of action against P. aeruginosa (25, 26). However, information regarding metabolomes and SCF resistance and regarding *in vivo*- and *in vitro*-evolved antibiotic-resistant derivatives from a parent strain is not available. The present study showed that although some metabolic differences are detected, most metabolic similarities are determined as characterized features of SCF resistance between AP-R_CLIN-EVO_ and AP-R_LAB-EVO_, where the downregulated fumaric acid, lactic acid, ornithine, and glucose are identified as biomarkers. Among the four biomarkers, fumaric acid and ornithine belong to the first (alanine, aspartate, and glutamate metabolism), second (arginine biosynthesis), and/or third (fourth) (the TCA cycle) impactful metabolic pathways and related to NO biosynthesis. Therefore, NO synthesis was used as a clue to explore SCF resistance in AP-R_CLIN-EVO_ and AP-R_LAB-EVO._

NO level that is related to antibiotic resistance has been demonstrated in bacteria, including P. aeruginosa (27, 28). Although early studies suggested that the ability of an NO donor to protect P. aeruginosa biofilms against aminoglycosides by blocking the energy-dependent phases of the drug uptake was at the heart of antibiotic resistance (27, 28), recent investigations indicate that the NO donor synergizes with conventional antibiotics to kill P. aeruginosa through its interactions with cytochromes of the electron transport chain (29). The NO-based therapeutic exerts broad-spectrum antibacterial, antibiofilm, and mucolytic action in CF-relevant environments by nitric oxide-releasing chitosan oligosaccharides, which may promote bactericidal activity via electrostatic attraction to bacteria and more localized NO release (29). The NO donor sodium nitroprusside induces biofilm dispersal, which is more sensitive to tobramycin than control biofilm. Loss of *nirS*, encoding nitrite reductase generating metabolic NO through anaerobic respiration, does not disperse, while absence of *norCB*, encoding NO reductase, exhibited greatly enhanced dispersal (30). However, endogenous NO comes from the l-arginine–NO pathway and nitrite-dependent NO biosynthesis, but regulation for the endogenous NO level of nitrite-dependent NO biosynthesis in antibiotic resistance is largely unknown in bacteria. The present study showed that the reduced NO is a characteristic feature as a consequence of SCF resistance both *in vivo* and *in vitro*, which may be related to the l-arginine–NO pathway and nitrite-dependent NO biosynthesis. However, exogenous arginine did not promote the endogenous NO level, but nitrate and nitrite did, suggesting that the reduced NO level is attributable to the downregulation of nitrite-dependent NO biosynthesis. Further evidence indicated that reduction of electron donors from NADH provided by the P cycle is responsible for the reduced NO. Activation of the P cycle by fumarate promoted the NO level via providing more electrons from oxidization of NADH back to NAD^+^ for nitrite-dependent NO biosynthesis. Meanwhile, the increase in NO level was accompanied by the elevation of SCF-mediated killing. Similar SCF-mediated killing efficacy was detected among NADH-, nitrate-, and nitrite-induced potentiation, while the highest efficacy was determined in fumarate potentiation. The results suggest that the P cycle activated by fumarate promotes the other metabolisms to potentiate SCF-mediated killing except for the provided electrons. A piece of solid proof is that fumarate promotes the nitrite level. Interestingly, exogenous substrates nitrate and nitrite promoted expression of Cyt bc1 complex genes, suggesting a feedback of exogenous substrates nitrate and nitrite to the electrons required in nitrite-dependent NO biosynthesis. Therefore, the nitrite-dependent NO biosynthesis and the P cycle are mutual in promoting NO metabolism.

Notably, the similarities are dominant, but differences exist. Approximately half of metabolites (43.6%) are different between AP-R_CLIN-EVO_ and AP-R_LAB-EVO_, where more unique upregulated metabolites are detected in AP-R_CLIN-EVO_, while more unique downregulated metabolites are found in AP-R_LAB-EVO_. Furthermore, AP-R_CLIN-EVO_ has more unique biomarkers than AP-R_LAB-EVO_. These differences suggest more complex environments *in vivo* than *in vitro*.

Our ultimate goal is to metabolically reprogram the bacteria to an antibiotic-sensitive state. However, nitrite, nitrate, and sodium nitroprusside have the potentiation, but further studies are needed for clinical trial. Specifically, a mouse model should be established to test the efficacy and safety dose of these compounds in potentiating SCF. Furthermore, the pharmacokinetics of synergistic use of SCF and nitrite, nitrate, or sodium nitroprusside should be measured. Comparatively, sodium nitroprusside is more convenient since it has already been a clinical drug.

**FIG 9:**
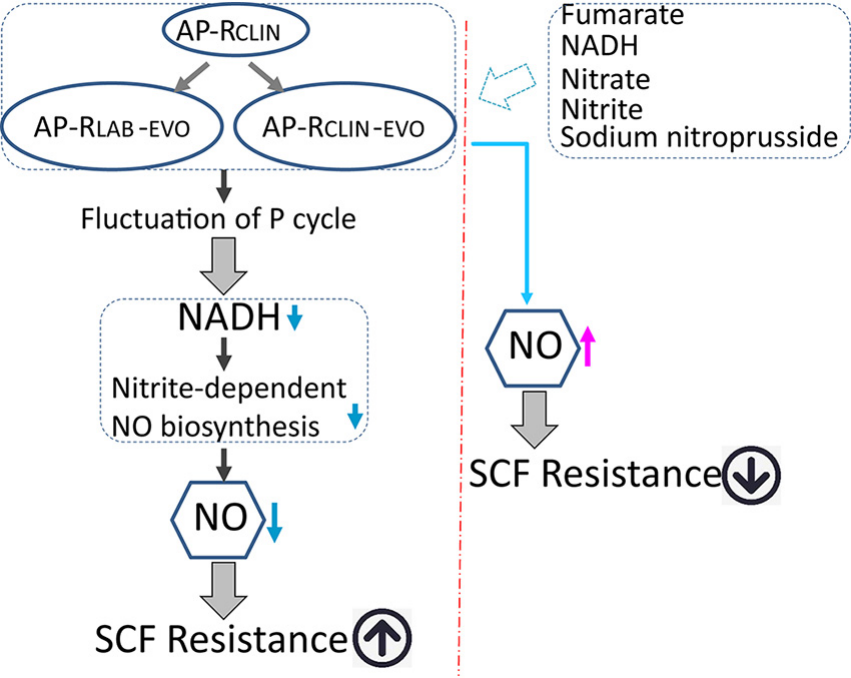
Diagram for a shared metabolic mechanism between AP-R_CLIN-EVO_ and AP-R_LAB-EVO_ to resist SCF.

The present study adopted GC-MS-based metabolomics to understand the global changes in metabolism and characterize the reduced NO as a consequence of SCF resistance in AP-R_CLIN-EVO_ and AP-R_LAB-EVO_ compared to AP-R_CLIN_. Further experiments demonstrated that inactivation of nitrite-dependent NO biosynthesis is responsible for the reduced NO, which is attributable to the reduced nitrite and NADH due to fluctuation of the P cycle. The fluctuated P cycle provides fewer electrons for nitrite-dependent NO biosynthesis and also affects the nitrite level, and thereby, the NO level is reduced. The conclusion is further supported by two events. First, exogenous fumarate promotes not only NADH but also nitrite. Equally, nitrate or nitrite synergizes expression of the nitrite-dependent NO biosynthesis gene and Cyt bc1 complex gene. Exogenous fumarate, NADH, nitrate, and nitrite as well as the NO donor sodium nitroprusside lead to co-occurrences of the elevated NO, and sensitivity to SCF supports that the reduced NO plays a role in SCF resistance in *in vivo*- and *in vitro*-evolved SCF-resistant strains from the same parent strain (Fig. 9). These results highlight the way to understand metabolic mechanisms of antibiotic resistance and explore metabolic modulation to combat bacterial pathogens.

## MATERIALS AND METHODS

### Bacterial strains and growth conditions.

Bacterial strains used in the current study were clinically isolated P. aeruginosa (AP-R_CLIN_) and its naturally (AP-R_CLIN-EVO_) and artificially (AP-R_LAB-EVO_) evolved strains (Table S1). These strains were cultured at 37°C, with shaking at 200 rpm in LB medium at an optical density at 600 nm (OD_600_) of 0.25 to 0.3, and then these cultures were reinoculated at a 1:1,000 dilution into 250-ml flasks and incubated for 16 h. The overnight bacteria were collected, washed three times with saline, and suspended in M9 medium [7 g/liter of K_2_HPO_4_, 3 g/liter of KH_2_HPO_4_, 1 g/liter of (NH_4_)_2_SO_4_, 0.1 g/liter of MgSO_4_, 0.588 g/liter of sodium citrate] to an OD_600_ of 0.2, when metabolites or/and antibiotics were added if desired. Then these bacterial cells were cultured at 37°C with shaking at 200 rpm for 6 h.

### Measurement of MIC and growth curve.

According to CLSI guidelines, the MICs of AP-R_CLIN_, AP-R_CLIN-EVO_, and AP-R_LAB-EVO_ were measured by antimicrobial susceptibility testing. In brief, overnight bacteria cultured in LB medium were diluted 1:100 in fresh LB broth and cultured at 37°C with shaking at 200 rpm to an optical density at 600 nm of 0.5. Then 10 μl of 5 × 10^6^ CFU/ml was added into each well of a 96-well microtiter polystyrene tray with 100 μl of a series of 2-fold dilutions of an antibiotic. The mixtures were incubated at 37°C for 16 h. MIC was defined as the lowest antibiotic concentration that inhibited visible growth. For growth curve, following the same dilution, these bacteria were cultured at 37°C with shaking at 200 rpm and monitored at 1, 2, 3, 4, 5, 6, 8, 12, 16, 20, 24, and 28 h through measurement of OD_600_. Three biological repeats were carried out.

### Illumina sequencing and bioinformatics analysis.

As previously described, a high-throughput method was used to analyze genotypic differences between AP-R_CLIN_, AP-R_CLIN-EVO_, and AP-R_LAB-EVO_ utilizing whole-genome resequencing data generated by Illumina sequencing (10). Raw reads were quality checked, and mutant-specific mutations were identified. Then the mutations were annotated and the hits and obtained coordinates were reviewed from the P. aeruginosa PAO1 reference, which has a complete genome and annotation information published in NCBI database. With those transformed positions from P. aeruginosa PAO1, the mutations were finally annotated by Annovar.

### Confirmation of mutation sites based on PCR sequencing.

For confirmation of mutation sites, overnight bacteria from three colonies of AP-R_CLIN_ and 20 colonies of AP-R_CLIN-EVO_ and AP-R_LAB-EVO_ were washed 3 times with double-distilled water (ddH_2_O). The bacteria were lysed by the boiling method, and the supernatant was centrifuged at 12,000 × *g* to obtain the genome. A PCR system with 50 μl was used: 2 μl (10 μM) of forward primer, 2 μl (10 μM) of reverse primer, a 0.5-μl mixture of EasyTaq DNA polymerase, 5 μl of 10× *Taq* buffer, 4 μl (2.5 mM each) of deoxynucleoside triphosphate (dNTP) mix, and 34 μl of ddH_2_O. Cycling conditions were 94°C for 5 min and 30 cycles of 94°C for 1 min, 58°C for 30 s, and 72°C for 1 min, followed by a final extension at 72°C for 10 min. Gene-specific primers used for PCR are shown in Table S2. All PCR products were analyzed by agarose gel electrophoresis and sent for sequencing (Guangzhou IGE Biotechnology Ltd., China). The sequences were compared at https://www.ebi.ac.uk/Tools/msa/clustalo/.

10.1128/mSystems.00732-21.5 TABLE S2 Primers for mutation sites based on PCR sequencing. Download Table S2, DOCX file, 0.01 MB. Copyright © 2021 Kuang et al. 2021 Kuang et al. https://creativecommons.org/licenses/by/4.0/ This content is distributed under the terms of the Creative Commons Attribution 4.0 International license.

### Preparation for metabolomic samples and GC-MS analysis.

Metabolomic samples were collected as previously described (11). In brief, overnight bacteria were diluted 1:100 in 50 ml of fresh LB medium and cultured at 37°C with shaking at 200 rpm until the culture reached an OD_600_ of 1.0. Then 10 ml of bacterial cells was collected by centrifugation at 5,000 × *g* for 5 min and washed 3 times with saline. The cells were immediately quenched with −80°C precooled methanol (high-performance liquid chromatography [HPLC] grade). Resuspension of the quenched cells was sonicated for 10 min (200-W total power with 35% output, 2-s pulse, and 3-s pause over ice) when 10 μl of 0.1-mg/ml ribitol (Sigma) was added as the internal standard. Supernatant was separated by centrifugation at 4°C and 12,000 × *g* for 10 min and placed in a 37°C vacuum centrifuge dryer (Labconco, USA) to evaporate the methanol. 80 μl of 20-mg/ml methoxyamine-pyridine hydrochloride (Sigma-Aldrich) was added to the dried samples and the reaction was performed at 37°C and 200 rpm for 3 h. Subsequently, 80 μl of *N*-methyl-*N*-trimethylsilyltrifluoroacetamide (MSTFA; Sigma-Aldrich) was added and the reaction was performed at 37°C and 200 rpm for 30 min. The GC-MS data were detected with an Agilent 7890A GC equipped with an Agilent 5975C VL MSD detector (Agilent Technologies). Samples of 1 μl were injected into a 30-m by 250-μm (internal diameter [i.d.] by 0.25-μm DBS-MS column. The initial temperature of the GC oven was held at 85°C for 5 min, followed by an increase to 270°C at a rate of 15°C/min, and then held for 5 min. Helium was used as the carrier gas, and flow was kept constant at 1 ml/min. The MS was operated in a range of 50 to 600 *m/z*. Four biological repeats with two technical replicas were prepared for each sample.

### Analysis of metabolomic data.

Statistical analysis was performed as described previously. In a concise manner, the mass fragmentation spectrum was analyzed by XCalibur software (Thermo Fisher; version 2.1) to identify compounds by matching data with the National Institute of Standards and Technology (NIST) library and NIST MS search 2.0 program. The data were normalized based on total amount correction and standardized data containing metabolites, retention times, and peak areas and used for further metabolomics analysis. Software IBM SPSS Statistics 19 was used to analyze significant difference of the standardized data, and metabolites with differences were selected (*P* value < 0.05). R software (*R* × 64 3.6.1) was used for cluster analysis. Principal-component analysis (PCA) and S-plot analysis were performed by SIMCA-*P* + 12.0 software (version 12; Umetrics, Umea, Sweden), and the metabolic pathway was done with MetaboAnalyst 4.0 enrichment. Interactive Pathways (iPath) analysis was carried out by iPath3.0 (https://pathways.embl.de/).

### Measurement of nitric oxide.

NO measurement was performed according to the total NO assay kit by the nitrate reductase method (Nanjing Jiancheng Bioengineering Institute; A012-1). Bacteria were cultured in M9 medium with or without metabolites at 37°C with shaking at 200 rpm for 6 h. Cells were collected and washed three times with saline. The cells were suspended in saline and adjusted to an OD_600_ of 1.0. An aliquot of 30 ml of cells was collected and transferred to a 1.5-ml centrifuge tube. The cells were resuspended with 0.6 ml of saline and disrupted by sonic oscillation for 7 min (200-W total power with 35% output, 2-s pulse, and 3-s pause over ice). Following centrifugation at 12,000 × *g* for 10 min at 4°C, supernatants were collected. The protein concentration of the supernatant was quantified with a bicinchoninic acid (BCA) protein concentration determination kit (Beyotime; P0009). Then, 500 μl of 4-mg/ml proteins was mixed with 400 μl of buffer I at 37°C for 60 min. The reaction solution was added to 300 μl of buffer II, vortexed and mixed well for 30 s, and then sedimented at 25°C for 40 min. Supernatant was obtained by concentration at 1,600 × *g* for 10 min. An aliquot of 800 μl of supernatant was mixed with 600 μl of color developer and sedimented at 25°C for 10 min. Absorbance was measured at 550 nm with a cuvette with a 0.5-cm optical path. The same volume of protein buffer and 0.1-mmol/liter standard samples were used as a blank control and a reference for concentration calculation, respectively. NO concentration (in micromoles per gram) was calculated with the following formula: NO concentration = (experiment group OD value – blank OD value)/(standard sample OD value – blank OD value) × standard sample concentration/protein concentration.

### Measurement of enzyme activity.

For measurement of pyruvate dehydrogenase (PDH), alpha-ketoglutarate dehydrogenase (KGDH), succinate dehydrogenase (SDH), and malate dehydrogenase (MDH) activities, bacterial cells were suspended in 1× phosphate-buffered saline (PBS; pH 7.4) and adjusted to an OD_600_ of 1.0. An aliquot of 50 ml of cells was collected and transferred to a 1.5-ml centrifuge tube. The cells were resuspended with 1 ml of 1× PBS and disrupted by sonic oscillation for 6 min (200-W total power with 35% output, 2-s pulse, and 3-s pause over ice). Following centrifugation at 12,000 × *g* for 10 min at 4°C, supernatants were collected. The protein concentration of the supernatants was quantified with a BCA protein concentration determination kit (Beyotime; P0009). Then, 300 μg of proteins was used for determination of enzyme activity. For PDH and KGDH measurement, the reaction mixture contained 0.15 mM 3-(4,5-dimethyl-2-thiazolyl)-2,5-diphenyl-2H-tetrazolium bromide (MTT), 2.5 mM MgCl_2_, 6.5 mM phenazine methosulfate (PMS), 0.2 mM thiamine PP_i_ (TPP), and 80 mM sodium pyruvate/alpha-ketoglutaric acid potassium salt, with water added to 200 μl. For SDH and MDH measurement, the reaction mixture contained 0.15 mM MTT, 2.5 mM MgCl_2_, 13 mM PMS, and 80 mM sodium succinate/sodium malate, with water added to 200 μl. All of the reaction mixtures were incubated at 37°C, for 5 min for MDH/PDH/KGDH and for SDH for 10 min. Finally, they were measured at 562 nm for colorimetric readings. Light was avoided in all reactions. Experiments were repeated in at least three independent biological replicates.

### Quantification of nitrite.

Nitrite quantification was performed using a commercial kit (Nanjing Jiancheng Bioengineering Institute; A038-1-1). In brief, after incubation in M9 with or without metabolites at 37°C with shaking at 200 rpm for 6 h, bacterial cells were suspended in saline and adjusted to an OD_600_ of 1.0. An aliquot of 60 ml of cells was collected and transferred to a 1.5-ml centrifuge tube. The cells were resuspended with 0.8 ml of saline and disrupted by sonic oscillation for 15 min (200-W total power with 35% output, 2-s pulse, and 3-s pause over ice). Aliquot of 800 μl (10 mg/ml) of proteins were reacted with 1,200 μl of buffer reagent at 25°C for 10 min. Supernatant was obtained at 1,600 × *g* for 10 min. An aliquot of 800 μl of supernatant and 400 μl of color developer were mixed and sedimented at 25°C for 15 min. Absorbance was measured at 550 nm with a cuvette with a 0.5-cm optical path. The same volume of saline was used as a blank control group, and 100-μmol/liter standard samples were used as a reference for concentration calculation. Finally, nitrite content (in micromoles per liter) was calculated with the following formula: nitrite content = (experimental group OD value – blank OD value)/(standard sample OD value – blank OD value) × standard sample content × protein sample dilution factor.

### Determination of NADH.

NADH was measured with the EnzyChrom NADH assay kit (BioAssay Systems) according to the manufacturer’s instructions. Briefly, after incubation in M9, with or without metabolites at 37°C with shaking at 200 rpm for 6 h, bacterial cells were collected and washed three times with sterile saline. Then the cells were diluted to an OD_600_ of 1.0 with saline and 2 ml of the cells was harvested at 12,000 × *g* for 3 min. Three parallel samples of the resulting cells were resuspended uniformly in NADH extraction buffer and vortexed for 15 s. Then the samples were incubated in 60°C water for 5 min. Assay buffer and opposite extraction buffer were added. After vortexing for 10 s and centrifugation at 19,000 × *g* for 5 min, the supernatant of samples was then diluted two times and used for measurement according to the manufacturer’s instructions.

### Analysis of gene expression.

For analysis of gene expression, quantitative real-time PCR (qRT-PCR) was completed as previously described (31). In brief, after incubation in M9 with or without metabolites at 37°C with shaking at 200 rpm for 6 h, cells were collected and adjusted to an OD_600_ of 1.0. An aliquot of 1 ml was transferred to a 1.5-ml QSP centrifuge tube. Supernatant was removed by centrifugation at 12,000 × *g* for 3 min, and the pellet was used for total RNA isolation using TRIzol reagent (Invitrogen Life Technologies). Total RNA (1 μg) was used as the template for qRT-PCR by a PrimeScript RT reagent kit with gDNA Eraser (TaKaRa, Japan) according to the manufacturer’s instructions. The primers used for qPCR are shown in Table S3; the 16S rRNA gene served as an internal control. The qRT-PCR was performed in 384-well plates with a total volume of 10 μl. The reaction mixtures were run on a LightCycler 480 system (Roche, Germany). The cycling parameters were as follows: 95°C for 30 s to activate the polymerase and 40 cycles of 95°C for 5 s and 58°C for 30 s. Fluorescence measurements were performed at 75°C for 1 s during each cycle. Cycling was terminated at 95°C with a calefactive velocity of 0.11°C s^−1^ to obtain a melting curve. Data are shown as relative mRNA expression compared with the control group with the endogenous reference 16S rRNA gene.

10.1128/mSystems.00732-21.6 TABLE S3 Primers for qRT-PCR. Download Table S3, DOCX file, 0.01 MB. Copyright © 2021 Kuang et al. 2021 Kuang et al. https://creativecommons.org/licenses/by/4.0/ This content is distributed under the terms of the Creative Commons Attribution 4.0 International license.

### Antibiotic bactericidal assay.

The antibiotic bactericidal assay was carried out as previously described (32, 33). As mentioned above, after culture in M9 with or without metabolites and/or antibiotics, bacteria were incubated at 37°C with shaking at 200 rpm for 6 h. To determine CFU per ml, 100-μl samples were 10-fold serially diluted and an aliquot of 10 μl of each dilution was spotted onto the LB agar plates and cultured at 37°C for about 11 h. The percent survival was determined by dividing the CFU obtained from the treated sample by the CFU obtained from the control.

### Data availability.

The genome sequence data utilized in this study have been deposited at the National Center for Biotechnology Information (NCBI) Sequence Read Archive (SRA) under the BioProject number PRJNA753974.
