# Supplementary figures and images for: Inactivation of Nitrite-Dependent Nitric Oxide Biosynthesis Is Responsible for Overlapped Antibiotic Resistance between Naturally and Artificially Evolved Pseudomonas aeruginosa
Source: mSystems. 2021 Sep 21;6(5):e00732-21. doi: 10.1128/mSystems.00732-21 (PMC8547483; doi:10.1128/mSystems.00732-21)

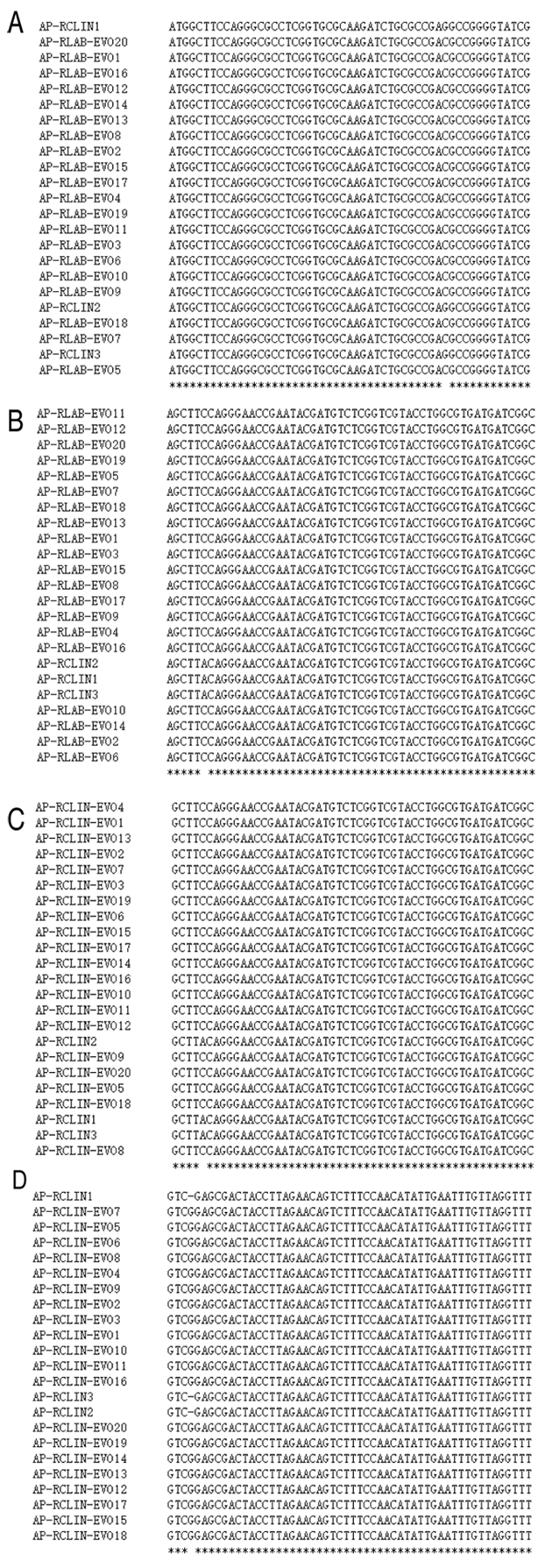

Supplement: FIG S1 [file msystems.00732-21-sf001.tif]

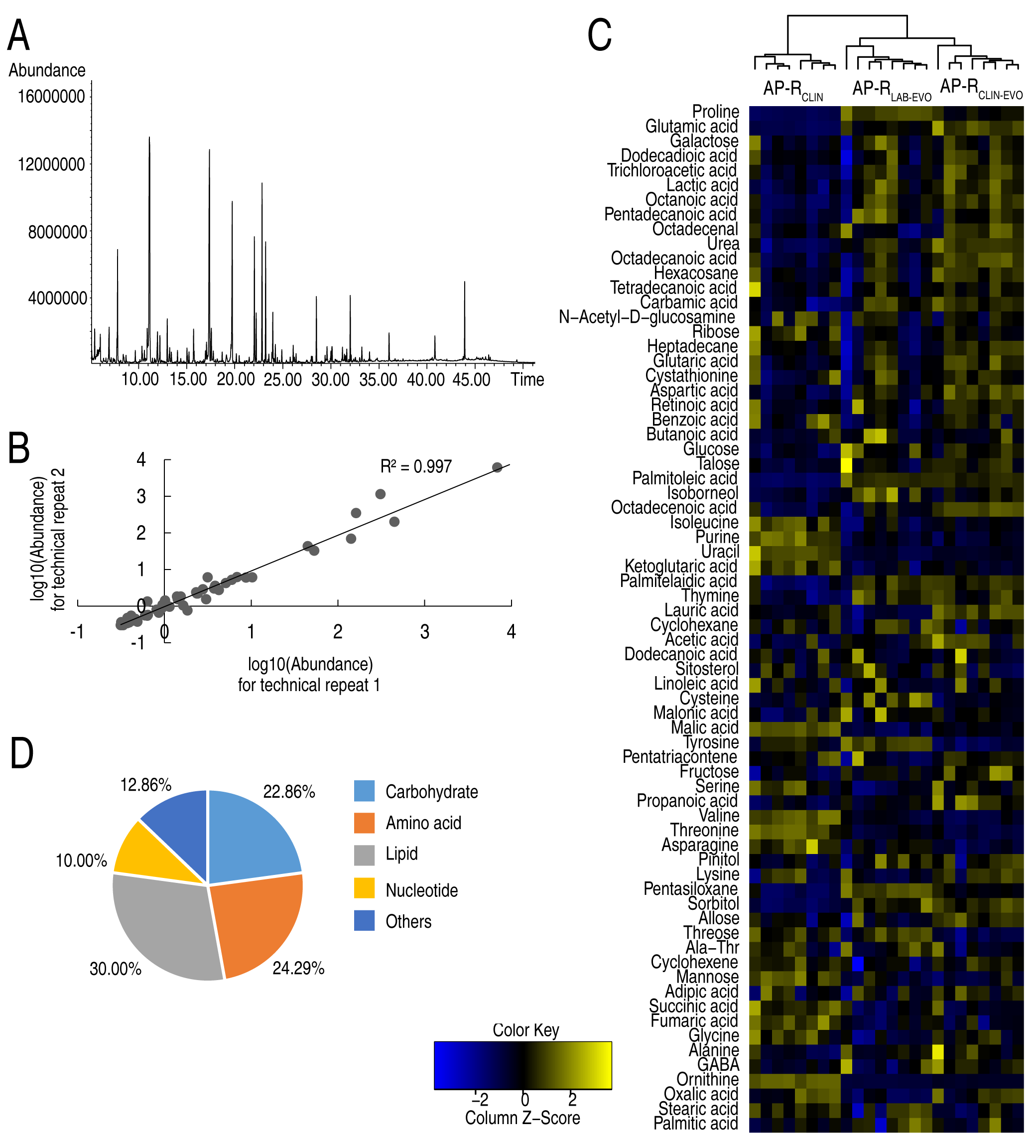

Supplement: FIG S2 [file msystems.00732-21-sf002.tif]

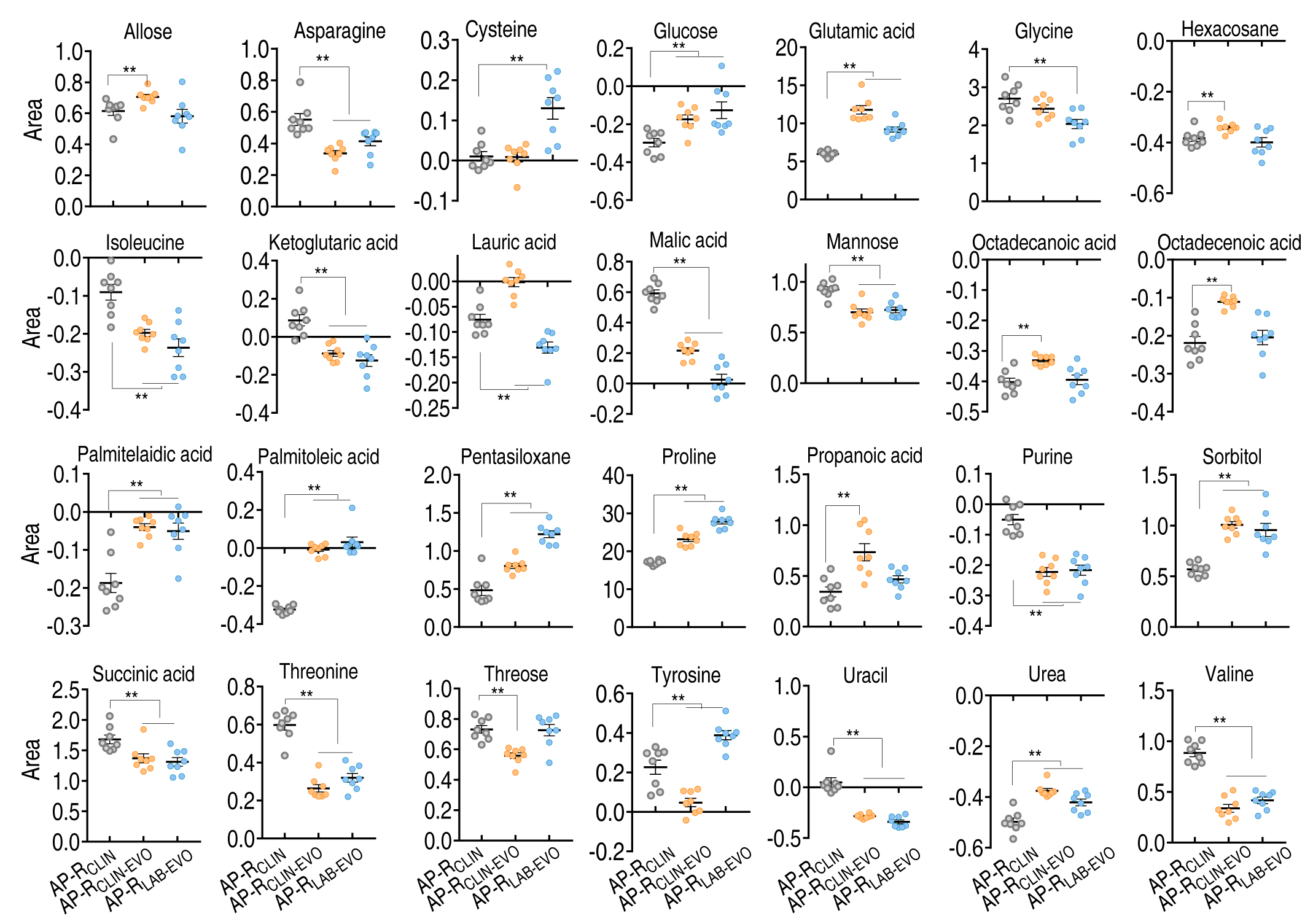

Supplement: FIG S3 [file msystems.00732-21-sf003.tif]
