# Supplementary material for: Inactivation of Nitrite-Dependent Nitric Oxide Biosynthesis Is Responsible for Overlapped Antibiotic Resistance between Naturally and Artificially Evolved Pseudomonas aeruginosa
Source: mSystems. 2021 Sep 21;6(5):e00732-21. doi: 10.1128/mSystems.00732-21 (PMC8547483; doi:10.1128/mSystems.00732-21)
Supplement: TABLE S1 [file msystems.00732-21-st001.docx]

**Table S1 Strains isolated from a patient**

| **Patient** | **Strain** | **Antibiotics** | **Collection day from admission date** |
| --- | --- | --- | --- |
| A | AP-_CLIN_ | **SCF** 3g, q12h, 3 days | 3 |
|  | AP-R_CLIN-EVO_ | **SCF** 3g, q12h, 3 days | 6 |
|  | | | |
