# Supplementary material for: Inactivation of Nitrite-Dependent Nitric Oxide Biosynthesis Is Responsible for Overlapped Antibiotic Resistance between Naturally and Artificially Evolved Pseudomonas aeruginosa
Source: mSystems. 2021 Sep 21;6(5):e00732-21. doi: 10.1128/mSystems.00732-21 (PMC8547483; doi:10.1128/mSystems.00732-21)
Supplement: TABLE S2 [file msystems.00732-21-st002.docx]

**Table S2 Primers for mutation sites based on PCR sequencing**

| **Gene** | **Primer sequence (5'-3')** |
| --- | --- |
| *tetR*-F | ATGAACCAGGAAGTGCG |
| *tetR*-R | CCCAGACTCCAGCCACA |
| DDE-F | ATCTGGACGAACTGAACGCT |
| DDE-R | GCACTCCGTCGGGGTAG |
| *cyt* B6-F | AAGACCGAATCGCAGGCAA |
| *cyt* B6-R2 | AGGGGCGGAAACGCAACC |
