# Supplementary material for: Inactivation of Nitrite-Dependent Nitric Oxide Biosynthesis Is Responsible for Overlapped Antibiotic Resistance between Naturally and Artificially Evolved Pseudomonas aeruginosa
Source: mSystems. 2021 Sep 21;6(5):e00732-21. doi: 10.1128/mSystems.00732-21 (PMC8547483; doi:10.1128/mSystems.00732-21)
Supplement: TABLE S3 [file msystems.00732-21-st003.docx]

**Table S3 Primers for qRT-PCR**

| **Gene** | **Primer sequence (5'-3')** |
| --- | --- |
| *pckA*-F | CCGATGACAGCCTGACCCA |
| *pckA*-R | ACCGAAGCAGGTGGAGAAGG |
| *aceE*-F | CAGTTCCCCACCGTATCCA |
| *aceE*-R | CGAAGATCAGGTTGTCGAGTTT |
| *aceF*-F | GGCCCGCAACAAGAAGCT |
| *aceF*-R | CTTGCCGTCCCATACCG |
| *gltA*-F | CGTGATGTGCGGCGTGA |
| *gltA*-R | GGTCGGCGTGGAGAATGA |
| *icd*-F | CCGAGGAAATGGGCGTCA |
| *icd*-R | CGACTTCGTAGCCCCAATC |
| *idh*-F | GAACCCGAACAACGGTATCTC |
| *idh*-R | CCGCCTTGGTGTCTTTCTGCT |
| *sucB*-F | GTGGCGTGTTCGGTTCCCT |
| *sucB*-R | ATCAGACGGTGGTCGTAGGA |
| *sdhA*-F | GAAGAAGTCGCTCCGCTCAA |
| *sdhA*-R | GAATGCCTGGCTCTTGTCG |
| *sdhC*-F | CCGTGAATAGCAAACGACCTG |
| *sdhC*-R | GGCAATACCGAGGAACAGGA |
| *sdhD*-F | TTCCTGCTGGGCTACCTCATT |
| *sdhD*-R | CAGGGTCAACAGGCTGAAGAT |
| *fumC2*-F | GGACCGAAACCGATAGCC |
| *fumC2*-R | GCGGGAAGTGTTCGTCGTG |
| *mqoB*-F | CCCCTATGCCGGTTTCTCC |
| *mqoB*-R | CGGGTCAGGTCCATGTTGT |
| *cyt* 1-F | GCTGCAAGCAGGTACAGGTGG |
| *cyt* 1-R | GCGAATACGAAGAAGAAGGCC |
| *cyt* b-F | AACCAGTTCAAGACCCCCG |
| *cyt* b-R | AGCCAGCCCTTGTAGCGGA |
| *ISP*-F | GCGGCAAGCCTGTATTCATC |
| *ISP*-R | CGTGGCAAGGGCAGAAGT |
| napA-F | CTTCGTCCTCTGGGGCTCG |
| *napA*-R | GATGATGTGGTTGGCGATG |
| *napB*-F | CAGCATCGTCGGCTATCGC |
| *napB*-R | ATCACCGCCTGGGTTCGC |
| *nirS*-F | ACGGCGACAGCAAGAAGA |
| *nirS*-R | GGTCCTCGTAGCCCTTGAA |
| *norB*-F | CGCCAAACCCTACTTCGTGT |
| *norB*-R | TCGCAGTCGCTCTCCTCC |
| *nosZ*-F | TTCATCATCCCCCATCCCA |
| *nosZ*-R | AACCTGCCGCTGTAGTCCA |
| *narH*-F | TCGGGTTGAGCGTGGAGCA |
| *narH*-R | ACAGGTTGACGGCGGAGTT |
| *nirB*-F | CGGCTCGCTCCCCTTCATCC |
| *nirB*-R | GGTGTTCAGGCGGAAGCGGA |
